# Autologous stem cell transplantation in refractory Crohn’s disease – low intensity therapy evaluation (ASTIClite): study protocols for a multicentre, randomised controlled trial and observational follow up study

**DOI:** 10.1186/s12876-019-0992-2

**Published:** 2019-05-31

**Authors:** John A. Snowden, Chris Hawkey, Daniel Hind, Lizzie Swaby, Katie Mellor, Richard Emsley, Laura Mandefield, Ellen Lee, Manuela Badoglio, Emmanuelle Polge, Myriam Labopin, John Gribben, A. Graham Pockley, Gemma A. Foulds, Alan Lobo, Simon Travis, Miles Parkes, Jack Satsangi, Diana Papaioannou, James O. Lindsay, on Behalf of the Autologous Stem Cell Transplantation In Refractory CD - Low Intensity Therapy Evaluation Study Investigators, Liam Whitby, Yash Mahida, Gordon Moran, Sree Subramanian, Peter Irving, Shahida Din, Jenny Byrne, Ben Uttenthal, Amit Patel, Peter Johnson, Majid Kazmi, Andy Peniket, Sergio Rutella, Mike Bradburn, Amanda Loban, the European Society for Blood and Marrow Transplantation (EBMT) Autoimmune Diseases Working Party (ADWP)

**Affiliations:** 1 Department of Haematology, Sheffield Teaching Hospitals NHS Foundation Trust, Royal Hallamshire Hospital, Sheffield, UK; 2 Nottingham Digestive Diseases Centre, University of Nottingham, Nottingham, UK; 3 Clinical Trials Research Unit, ScHARR, University of Sheffield, Sheffield, UK; 4 Department of Biostatistics & Health Informatics, Institute of Psychiatry, Psychology & Neuroscience, King’s College London, London, UK; 5 European Society for Blood and Marrow Transplantation, Paris, France; 6 Centre for Haemato-Oncology, Barts Cancer Institute, Queen Mary University of London, Charterhouse Square, London, UK; 7 John van Geest Cancer Research Centre, School of Science and Technology, Nottingham Trent University, Nottingham, UK; 8 Department of Gastroenterology, Sheffield Teaching Hospitals NHS Foundation Trust, Royal Hallamshire Hospital, Sheffield, UK; 9 Translational Gastroenterology Unit, NIHR Oxford Biomedical Research Centre, Oxford University Hospitals NHS Foundation Trust, John Radcliffe Hospital, Oxford, OX3 9DU UK; 10 Department of Medicine, University of Cambridge, Cambridge, UK; 11 Centre for Immunobiology, Barts and the London School of Medicine and Dentistry, Blizard Institute, Queen Mary University of London, London, UK

**Keywords:** Crohn’s disease, Autologous stem cell transplant, Randomised controlled trial, Observational study

## Abstract

**Background:**

Intestinal inflammation in Crohn’s disease (CD) is caused by mucosal immune system reactivity to luminal antigen and results in debilitating symptoms, reduced quality of life, impaired work productivity and significant health care costs. Not all patients respond to conventional and biologic therapies, with chronic inflammation ensuing. Although surgical resection may be required, disease frequently returns and surgery may not be an option, or may be declined. Case reports suggest potential benefit after haematopoietic stem cell transplant (HSCT) for patients with refractory CD.

The ASTIC trial asked whether HSCT could cure CD. Few patients achieved the primary endpoint of clinical remission for 3 months, off all medication with no evidence of active disease, and there were a high number of adverse events (AEs) and serious adverse events (SAEs), including one patient death. However, beneficial effects were observed in some aspects of disease activity. The ASTIClite trial will investigate these potential benefits and safety using a lower intensity regimen than ASTIC.

**Methods:**

Ninety-nine participants will be recruited from secondary care IBD centres in the UK into a multicentre, randomised controlled trial (RCT, ASTIClite) and an observational follow-up, and randomised to autologous HSCT versus standard care (ratio 2:1).

The primary endpoint is treatment success at week 48, defined as mucosal healing without surgery or death. Secondary endpoints relating to efficacy, safety and mechanistic analyses will be evaluated at week 8, 14, 24, 32, 40 and 48.

Long-term safety of the low intensity HSCT regimen forms the primary endpoint for the EBMT follow-up study and will be assessed annually for 4–7 years.

**Discussion:**

ASTIClite will compare HSCTlite with standard care with respect to safety, efficacy and quality of life, and capture outcomes allowing findings to be generalised to current clinical practice in the UK. It will also provide significant mechanistic insights into the immunological consequences of HSCTlite and its impact on treatment outcomes. The observational follow-up will provide information, which is currently unavailable for this population.

**Trial registration:**

The ASTIClite RCT was registered on 31st October 2017 (ISRCTN17160440) and the EBMT follow-up study on 19th January 2018 (ISRCTN31981313).

## Background

Intestinal inflammation in Crohn’s disease (CD) is caused by mucosal immune system reactivity to luminal antigen and results in debilitating symptoms, reduced quality of life, impaired work productivity and significant health care costs [1]. CD accounts for 27,000 hospital admissions in the UK each year [2]. Biological medications account for the largest element of patient costs in both secondary and tertiary care [3] with anti-TNF therapy-related costs also having increased over the last 2 years [2].

Although many patients respond to conventional and biologic therapies, the National Institute for Health Research (NIHR) portfolio cohort trial, Personalised Anti-TNF Therapy in Crohn’s disease (PANTS) (UKCRN 14175 & 17,747) of 1610 CD patients commencing anti-TNF therapy reports primary non-response at week 14 in 23·8% (95% CI 21·4–26·2) and non remission at week 54 in 63·1%, (60·3–65·8) [4]. Second line therapy with the gut-selective integrin inhibitor vedolizumab and an antibody against p40 (IL-12 and IL-23), ustekinumab are approved by The National Institute for Health and Care Excellence (NICE) for patients with CD refractory to or intolerant of steroids and conventional immunomodulators. However, in one phase III trial, vedolizumab did not achieve its induction primary endpoint in patients previously exposed to anti-TNF [5]. In another phase III trial, vedolizumab was not more effective than placebo at week 6 among patients with refractory CD, with remission in approximately 30% of patients by week 10 [6]. Therefore, patients with treatment refractory CD face persistent symptoms related to disease activity in addition to the morbidity associated with chronic steroid exposure. Although surgery may be an option, this may result in a permanent stoma and is often declined. An option for patients with refractory CD is haematopoietic stem cell transplant (HSCT) for which case reports suggest exceptional benefit [7, 8].

### The ASTIC trial

Under the European Society for Blood and Marrow Transplantation (EBMT) Autoimmune Diseases Working Party (ADWP), a randomised controlled trial, Autologous Stem cell Transplantation for Crohn’s Disease (ASTIC) was designed to answer the following questions; (i) does HSCT ‘cure’ CD and (ii) is any observed benefit derived from the cyclophosphamide or the stem cell transplant? [9]. For this, eligible patients underwent high dose (4 g/m^2^) cyclophosphamide / G-CSF mobilisation and were then randomised to immediate HSCT or conventional care for 1 year. Few patients in either group achieved the primary endpoint of clinical disease remission for 3 months, off all medication, with no evidence of active disease on imaging and endoscopy. In retrospect, this primary endpoint was more ambitious than that which had been used in any other trial published in this disease area. In addition, it may not be in a patient’s interest to have a protocolled withdrawal of therapy to meet the primary endpoint (off all therapy), given that it is known patients can relapse after HSCT and respond to therapies to which they were previously refractory. Finally, there were a high number of adverse (AEs) and serious adverse events (SAEs) that were not clinically acceptable, and one patient death. Consequently, HSCT is rarely used for patients with refractory CD in the UK.

However, ASTIC did demonstrate significant benefits in endpoints that are more traditional for therapeutic trials in this area and remain of clinical importance and relevance to patients, such as steroid-free clinical remission, mucosal healing and quality of life [9]. After the primary endpoint, patients in the control arm underwent HSCT. Subsequent analysis of the impact of HSCT in all patients in the ASTIC program reported that 50% of patients achieved regression of all endoscopic ulceration at 1 year. In addition, clinical factors that predict treatment success or AEs were identified [10]. A report by the EBMT in 2018, suggested that the retrospective analysis of HSCT for CD in multiple European centres, autologous HSCT appeared safe and effective in refractory CD, but that further trials are needed [11].

### Risks and benefits

The incidence of CD is increasing, particularly in young adults who may live with their disease for many decades [1]. Patients with refractory CD suffer impaired quality of life and disease- or treatment-related morbidity. In addition, refractory CD is associated with a heavy burden of direct health care costs including disease assessment, outpatient care, inpatient care, intravenous nutrition, surgery and medication costs [2, 3]. Biological therapies (both licensed and in phase III trials) are of a lower efficacy in treatment-experienced patients and have high acquisition costs. In the absence of an effective alternative, these patients are likely to be exposed to a sequence of expensive therapies that deliver a diminishing potential for benefit and increasing risk of harm.

The ASTIC trial [9] reported a high burden of AEs and one death. Subsequent specialist review suggested that the high dose cyclophosphamide used at both mobilisation and conditioning may have been a factor for many of the mobilisation-related infectious AEs [12, 13]. Research also highlighted the importance of supportive care in reducing the incidence of SAEs [14]. Analysis of the entire ASTIC cohort using more traditional endpoints demonstrates a significant benefit of this therapy at 1 year. Importantly, this analysis and previous studies have suggested that HSCT appears to restore responsiveness to anti-TNF therapies to which patients were previously refractory [8, 10]. Furthermore, a recent single centre cohort study with long-term clinical and endoscopic follow-up showed benefit extending to 5 years. Finally, reduced intensity mobilisation and conditioning regimens have been associated with lower morbidity in malignant and autoimmune disease [14–17].

### ASTIClite

ASTIClite is a multicentre, parallel group, randomised controlled trial designed to evaluate the efficacy of HSCTlite at inducing regression of intestinal ulceration in patients with refractory CD compared with standard care.

## Rationale for the study

Many specialists feel it would be inappropriate to interpret the complex ASTIC clinical trial with the simple message that HSCT is not effective for refractory CD for the following reasons [12, 13]:

1. The primary endpoint was more ambitious than in any other CD trial and the low frequency of patients that achieved it suggests that the trial was underpowered;
2. Both control and intervention group received cyclophosphamide at the relatively high dose of 4 g/m^2^ for mobilisation of peripheral blood stem cells (PBSCs), which had significant short-term beneficial impact on disease activity;
3. No maintenance therapy was used after HSCT in patients with recurrent disease;
4. HSCT achieved statistically significant differences over control for secondary endpoints including clinical remission and endoscopic disease activity;
5. Combined uncontrolled data of all transplanted patients shows a striking reduction in clinical and endoscopic disease activity at 1 year [10];
6. The high dose of cyclophosphamide used increased AEs [12, 13];
7. Reduced intensity HSCT regimens and enhanced supportive care have reduced HSCT morbidity [14, 18].

ASTIClite will therefore assess the clinical efficacy and long-term impact of low-dose cyclophosphamide/G-CSF mobilisation with reduced intensity conditioning in patients with active CD refractory to biologic therapies. Embedded mechanistic studies will assess the timeline of response, immune reconstitution after HSCT and the mechanism by which HSCT alleviates disease and restores anti-TNF responsiveness in this previously refractory group.

## Aims

The main aims of this study are to assess whether stem cell mobilisation with low-dose cyclophosphamide (1 g/m^2^) and G-CSF followed by autologous transplantation with a reduced intensity (‘HSCTlite’) conditioning regimen (fludarabine 125 mg/m^2^, cyclophosphamide 120 mg/kg and rabbit ATG 7.5 mg/kg) is safe and effective in inducing regression of intestinal ulceration in patients with refractory CD, when compared with standard care.

An observational follow-up study through the EBMT will assess the long-term benefit and safety of HSCTlite for a minimum of a further 4–7 years after the stem cell transplant. The follow-up will also investigate the burden of disease without a transplant, although no formal comparison between groups is planned. Patients in the control group who undergo HSCTlite after the end of ASTIClite will continue with annual follow up in the follow-up study.

This multi-centre RCT has been designed to have direct clinical applicability in the management of refractory CD in the UK following completion.

The objectives of the ASTIClite RCT are:

- Primary objective
  - To assess the efficacy of HSCTlite compared to standard care at inducing regression of intestinal ulceration in patients with refractory CD at week 48;
  - To investigate the long-term benefit and safety of HSCTlite using registry-based follow up for a minimum of a further 4–7 years after the stem cell transplant.
- Secondary objectives
  - To assess the impact of HSCTlite on clinical disease activity and quality of life compared to standard care.
- Safety objectives

The safety objectives in this study will initially be assessed as part of the embedded pilot study. The Data Monitoring and Ethics Committee (DMEC) will assess whether low-dose cyclophosphamide and G-CSF is a safe and effective mobilisation regimen for patients with refractory CD. Ongoing monitoring of toxicity of chemotherapy using the National Cancer Institute Common Terminology Criteria for Adverse Events (NCI CTCAE) will continue throughout the trial.

- Exploratory objectives
  - The safety and efficacy of re-introducing anti-TNF therapy in patients who demonstrate endoscopic disease recurrence at week 24 after HSCTlite
- Mechanistic objectives

HSCT is thought to induce regression of autoimmune diseases by altering the diversity of the T cell receptor repertoire and generating functional renewal of regulatory T cells and other immunoregulatory mechanisms that establish and maintain peripheral immune tolerance [19]. Neither the mechanism of action nor the time-course of response in CD is known. The mechanistic objectives in this study are:

- Intestinal MRI will be performed to determine the early impact of HSCT on mucosal disease at week 4;
- Immune profiling of peripheral blood and mucosal biopsies will:
  - ◦ Characterise immune re-constitution after HSCT, assess the impact of HSCT on disease activity and interrogate relationships between parameters of immune reconstitution and disease activity;
  - ◦ Assess immunological events that precede disease recurrence after HSCT;
  - ◦ Assess the mechanism of restoration of responsiveness to anti-TNF therapies

Serum will be stored for future immunological studies, including an assessment of response to vaccination after HSCT.

## Methods/design

### Trial design

The study is a parallel-group, controlled trial that will randomise eligible patients to low-dose cyclophosphamide and G-CSF mobilisation and reduced intensity conditioning for HSCT versus standard care (in the ratio 2:1).

Ninety-nine patients with clinical and endoscopic CD activity who are refractory to at least two classes of biologic therapy and in whom surgery is inappropriate or has been declined, will be recruited.

Participants will be recruited from eight NHS centres that have tertiary referral IBD clinics and HSCT will be carried out in centres that are either JACIE accredited for allogeneic HSCT, or for autologous HSCT if they have previous experience of autologous HSCT for CD (Bart’s Health, Sheffield, Nottingham, Oxford, Cambridge, Liverpool, Edinburgh and Guy’s and St Thomas’ (transplants to be carried out at King’s College Hospital for patients recruited at Guy’s and St Thomas’)) over a 3-year period, with a 1 year follow-up for the RCT.

Participants will also be invited to consent to long-term follow up through the EBMT Registry for up to a further 7 years.

An incorporated internal pilot will confirm whether the mobilisation regimen of 1 g/m^2^ cyclophosphamide delivers effective stem cell harvest without a flare up of CD activity. Ability to recruit to target will be assessed at month 10 of recruitment with STOP/GO criteria set at 60% of the anticipated recruitment at that time.

This protocol was written in accordance with SPIRIT [20].

### Participants

Potential participants will be identified by principal investigators and co-investigators through investigator sites or as a referral through neighbouring NHS Trusts. The trial will be discussed at local MDT meetings and publicised more widely through dissemination practices including press releases, websites and in journals. Any direct patient enquiries will be directed to their usual IBD team to seek a clinical referral to one of the participating centres for consideration for the trial.

Once identified, the potential participant will be invited to discuss the trial with the principal investigator, or delegated individual, and if they are interested will be invited to give their permission to be discussed with the trial MDT regarding suitability for screening.

### Eligibility, consent and randomisation

#### Inclusion criteria

A participant is eligible for the ASTIClite RCT if the following criteria are met:

1. Participant of any gender, aged between 18 and 60;
2. Participants must be willing and able to provide full informed consent;
3. Participants should be well nourished and of healthy weight in the opinion of the PI (typically BMI > 18.5);
4. Diagnosis of CD using colonoscopy, histology and/or radiology;
5. Disease duration of at least 6 months;
6. Disease distribution accessible to endoscopic assessment (jejuno-ileal, ileo-caecal, or colonic);
7. Active clinical CD activity with impaired quality of life at any time within 3 months prior to randomisation into the trial, as assessed by a gastroenterology clinician;
8. Participants will be refractory or intolerant to azathioprine, mercaptopurine or methotrexate;
9. Participants will be refractory or intolerant to at least two classes of biologic therapy (currently anti-TNF therapy, vedolizumab or ustekinumab), despite dose optimisation;
10. Participants where surgery is considered not appropriate or has been declined;
11. Endoscopic evidence of active disease in screening (SES CD ulceration sub-score of 2 or more in at least one segment). SES-CD will be used as standard for patients with disease in the ileum and/or colon. Should the disease only be proximal to the ileum, the SES-CD will still be used to score the relevant bowel segment;
12. Satisfactory screening assessment prior to HSCT (as per EBMT Autoimmune Disease Working Party (ADWP) recommendations [16]);
13. Willingness to discontinue all immunosuppressant medication after randomisation if allocated to HSCT arm;
14. Participants, who, in the opinion of the Trial Management Group (TMG), are fit enough to undergo treatment.

A participant is eligible for the EBMT follow up study if the following criteria are met:

1. Participants must have consented to take part in the ASTIClite RCT;
2. Participants must be willing and able to provide full informed consent, including sharing their data with the EBMT.

#### Exclusion criteria

A participant is not eligible for the ASTIClite RCT if any of the following criteria are met:

1. Diagnosis of ulcerative colitis or indeterminate colitis;
2. No evidence of active CD on screening endoscopic assessment;
3. Inability to assess for endoscopic active disease due to strictures;
4. Undrained perianal fistulae (patients with previous perianal disease or perianal disease adequately drained with a seton in situ are eligible);
5. Presence of undrained perianal sepsis on screening pelvic MRI;
6. Evidence of intra-abdominal sepsis on abdominal MRI;
7. Active or latent mycobacterial infection;
8. Prior exposure to Hepatitis B, Hepatitis C or HIV;
9. Evidence of an enteric or systemic infection;
10. Participant is currently pregnant, breastfeeding, or planning pregnancy within the duration of the study. Current pregnancy will be confirmed with a pregnancy test at screening assessment;
11. Unwilling to use adequate contraception (if appropriate) until at least 12 months after the last dose of study drug;
12. Contraindication to the use of cyclophosphamide, fludarabine, filgrastim or rabbit ATG;
13. Participants with significant medical co-morbidity that precludes HSCT adjudicated by the TMG;
14. Participants with significant psychiatric co-morbidity;
15. Significant language barriers, which are likely to affect the participant’s understanding of the study, or ability to complete outcome questionnaires;
16. Concurrent participation in another interventional clinical trial;
17. Participants who are not considered medically fit for HSCT defined by any of the following:
  - Renal: creatinine clearance < 40 ml/min (measured or estimated);
  - Cardiac: clinical evidence of refractory congestive heart failure, left ventricular ejection fraction < 45% by multigated radionuclide angiography (MUGA) or cardiac echo; uncontrolled ventricular arrhythmia; pericardial effusion with haemodynamic consequences as evaluated by an experienced echo cardiographer;
  - Hepatic: AST > two times the upper limit of normal;
  - Concurrent neoplasms or myelodysplasia;
  - Bone marrow insufficiency defined as neutropenia with an absolute neutrophil count < 1 × 10^9^/l, or thrombocytopenia with a platelet count < 50 × 10^9^/l, or anaemia with a haemoglobin < 80 g/l;
  - Uncontrolled hypertension, defined as resting systolic blood pressure > = 140 mmHg and/or resting diastolic pressure > = 90 mmHg despite at least 2 anti-hypertensive agents (subject to discussion at TMG);
  - Uncontrolled acute or chronic infection with HIV, HTLV – 1 or 2, hepatitis viruses or any other infection the investigator or TMG consider a contraindication to participation;
  - Other chronic disease causing significant organ failure, including established cirrhosis with evidence of impaired synthetic function on biochemical testing and known respiratory disease causing resting arterial oxygen tension < 8 kPa or CO_2_ tension > 6.7 kPa. FEV1/FVC < 50%. Patients not known to have respiratory disease need not have blood gas measurements.

A participant is not eligible for the EBMT follow-up study if any of the following criteria are met:

1. Significant language barriers, which are likely to affect the participant’s understanding of the study, or ability to complete outcome questionnaires.

#### Referral and consent

Potential participants will receive an approved participant information sheet and be given the opportunity to ask questions to the gastroenterology and haematology specialist teams prior to giving formal consent to participate.

All potentially eligible participants will need to sign a consent form to allow discussion on a case-by-case basis by a trial MDT panel for adjudication on eligibility. At least two gastroenterologist members of the panel, and one haematology member must agree on inclusion of the potential participant for them to progress to give full consent and undergo screening assessments.

If the MDT panel deem a potential participant ineligible, they will not be consented to the study, unless specific actions are requested such as further screening investigations, which subsequently confirm eligibility. In this case, patients will come back to the MDT for further discussion on eligibility, once the required actions are complete.

If the MDT agree that the potential participant appears eligible, the patient will be invited to give full written consent and proceed to screening investigations. Patients will have the opportunity to visit their local transplant centre and receive counselling from an independent clinician.

A medically qualified individual will confirm eligibility and provide clinical oversight for the consent process. In addition, patients will be asked if they wish to take part in the EBMT follow-up study.

A screening log maintained for each site will document all potential participants screened, whether they were recruited, and any reasons for non-recruitment where this information is available.

Once all screening investigations have been completed, the investigator will re-present the participant’s case with the trial MDT, confirming whether any information gathered during screening raises any concerns. The trial MDT will approve participants for randomisation if there are no concerns.

#### Randomisation

Once eligibility has been confirmed and baseline data recorded, participants will be centrally randomised to either the HSCTlite arm or usual care, in the ratio 2:1, using the CTRU online randomisation system (SCRAM). The randomisation schedule will be generated by the blinded trial statistician prior to the start of the study. The trial statistician will generate the schedule via SCRAM but will remain blinded to the allocation as they will not be able to access the schedule. Randomisation will be stratified by centre using permuted blocks of variable size to ensure that sufficient participants are allocated in the correct ratio each arm of the trial within each centre. Day 0 for the group allocated to receive usual care will be calculated as 49 days after the date of randomisation in order to align the length of time that both groups are within the trial, given the time taken to go through HSCTlite.

The patient’s GP will be informed of their participation in the study, including their trial allocation, as will their referring gastroenterologist (if appropriate).

## Interventions

### HSCTlite

Those participants randomised to receive HSCTlite will be asked to stop their current immunosuppressant medication. As mobilisation and conditioning are intensely immunosuppressive, additional immunosuppression is likely to be unnecessary and may pose additional risks. Corticosteroid medication will be continued, but weaned according to the protocol.

Participants will then commence mobilisation of peripheral blood stem cells (see Table 1), starting with an infusion of cyclophosphamide (1 g/m^2^). The mobilisation phase can be undertaken as either an inpatient or outpatient, depending on the sites’ local procedures. Mesna, an NIMP in this study, is given alongside cyclophosphamide to prevent haemorrhagic cystitis. Four days after cyclophosphamide infusion, participants will receive G-CSF (filgrastim or biosimilar) 5 μg/kg. From day 8, full blood and CD34^+^ cell counts will be monitored and once peripheral blood CD34^+^ cell levels exceed 10 × 10^6^/L, stem cells will be harvested (minimum 2.0 × 10^6^/kg CD34^+^ cells) and cryopreserved according to local protocols, allowing for 10% wastage through quality assessment.

**Table 1:** Timing of administration of IMP during mobilisation phase

Day	1	2	3	4	5	6	7	8	9	10	11	12
Cyclophosphamide 1 g/m^2^	✓											
G-CSF (filgrastim) 5 μg/kg					✓	✓	✓	✓	✓			
Mesna (dose as per local practice)	✓											
PB CD34 count								✓	✓	✓	✓	✓
Stem cell harvest								✓^a^	✓^a^	✓^a^	✓^a^	✓^a^

^a^Stem cell harvest is approximate, the day of this will depend on adequate CD34+ counts, as described above

Approximately 2–4 weeks after stem cell harvest, patients will be admitted to hospital for conditioning (see Table 2). IV fludarabine 25 mg/m^2^ is given on days − 6, − 5, − 4, − 3 and − 2, along with cyclophosphamide 60 mg/kg/day on days − 3 and − 2. Rabbit ATG (2.5 mg/kg) will be given on days − 3, − 2 and − 1. Methylprednisolone will be given alongside ATG, and then tapered according to local practice to cover ATG-related febrile and other reactions and protect against adrenal insufficiency. As with mobilisation, Mesna will be given alongside conditioning cyclophosphamide.

**Table 2:** Timing of administration of IMP during conditioning phase

Day	−6	−5	−4	−3	−2	−1	0	1	2	3	4	5
Fludarabine 25 mg/m^2^/day	✓	✓	✓	✓	✓							
Cyclophosphamide 60 mg/kg/day				✓	✓							
Mesna (dose as per local practice)				✓	✓							
Standard hydration (as per local practice)				✓	✓							
Rabbit ATG (Thymoglobulin; Genzyme) (2.5 mg/kg/day)				✓	✓	✓						
Methylprednisolone (1 mg/kg/day)				✓	✓	✓						
Stem cell reinfusion							✓					
G-CSF (filgrastim) (5 μg/kg/day)												✓ (continued until absolute neutrophil count > 1.0 × 10^9^/L for 2 days)

Stem cells will be reinfused on day 0, and G-CSF (filgrastim or biosimilar, 5 μg/kg/day) will start on day + 5 until the absolute neutrophil counts reach > 1.0 × 10^9^/L for 2 consecutive days.

Supportive care will follow local standard procedures at each centre and is at the discretion of the transplant physician, but should not include additional immunosuppression.

All transplants must be reported by the transplant centres to the EBMT database, as per standard practice. This will include any transplants that are received outside of the trial, while the EBMT follow-up study is in progress.

#### Standard care

Patients who are randomised to the standard of care arm will continue on conventional, biologic or nutritional therapy for their Crohn’s disease until assessment of the primary endpoint. There is no restriction on the treatment and supportive care that they can receive, which will be dictated by either the trial site or their regular clinical team dependent on preference. They will undergo the study related procedures and sample collection as detailed later in the protocol.

### Study procedures, sample collection and analysis

The study assessment schedule (Appendix) details the assessments required during the study. All participants will undergo these assessments at screening, baseline, week 8, week 14, week 24, week 32, week 40 week 48 regardless of the treatment arm to which they are randomised. Participants receiving the stem cell transplant will also have an MRI at week 4 to assess early response. A window of +/− 1 week is permitted for each study visit to take place. Concomitant medications and AEs will be recorded at all study visits.

### Screening and baseline assessments

Screening and baseline assessments will be performed after informed consent and can take place over several weeks. Screening and baseline assessments include:

1. Standard pre-HSCT workup, including chest X-ray and cardiac echo or MUGA scan (as per EBMT ADWP guidelines);
2. Assessment of clinical disease activity (CDAI and HBI);
3. Assessment of quality of life using patient completed questionnaires (IBDQ, IBD-Control, EQ5D, WPAI & Healthcare resource utilisation);
4. Endoscopic assessment of disease (endoscopic disease activity scored using SES CD) via colonoscopy, ileoscopy or balloon enteroscopy depending on location;
5. MRI small bowel to record disease activity (MaRIA score);
6. MRI pelvis in patients with previous perianal disease to exclude perianal sepsis;
7. Confirmation of eligibility by MDT;
8. Discussion about the impact of HSCT on fertility and referral to a fertility centre for semen or oocyte preservation if appropriate
9. Criteria for fitness for HSCT. Participants who meet one of more of these exclusion criteria, but in the opinion of the PI are medically fit enough to undergo HSCT, may be put forward to MDT for discussion about eligibility.

Assessment of disease activity (MaRIA score, SES CD and CDAI), and screening blood tests should occur within 8 weeks of randomisation. The patient will also be asked to complete a symptom diary for a week prior to assessment of the CDAI; this cannot be taken immediately preceding a colonoscopy and patients should finish the diary prior to starting bowel prep for colonoscopy.

### Procedures for assessing efficacy

#### Blood samples

In addition to locally processed standard blood tests, serum and whole blood (from which will be isolated peripheral blood mononuclear cells, PBMCs) will be collected from each participant at baseline, week 8, 14, 24, 32, 40 and 48 study visits.

Routine blood tests will be analysed in local laboratories. Samples for mechanistic studies will be shipped overnight to the John van Geest Cancer Research Centre at Nottingham Trent University, Nottingham for processing and analysis. All sample processing, analysis and reporting will be undertaken according to Good Clinical Laboratory Practice (GCLP) standards.

#### Ileo-colonoscopy

Ileo-colonoscopy will be performed at baseline, 24 and 48 weeks, according to local practice using a standard bowel preparation and conscious sedation. If the patient has a stoma, endoscopy through the stoma will be performed. Likewise, enteroscopy may be performed if the disease is limited to the small intestine.

Intestinal biopsies will be taken during the endoscopy/colonoscopy. Biopsies for routine histology will be sent to local laboratories in formalin. Biopsies for mechanistic analysis will be placed into RNAse reagent overnight and stored on site at − 80 °C. Samples will be sent to the John van Geest Cancer Research Centre every 3 months.

Videos of withdrawal from all endoscopies will be recorded. Eligibility for trial inclusion at baseline and the requirement for anti-TNF therapy at week 24 in the intervention group will be based upon local PI assessment of endoscopic assessment using SES CD. In patients where disease is proximal to the ileum, the SES CD will still be used to score the diseased bowel segment present. All videos will be centrally read using the SES CD by investigators blinded to site, treatment allocation and timing of procedure for analysis of primary and secondary outcomes.

#### Stool and stem cell samples for future research

Participants will also provide stool samples, which will be collected, frozen and stored for future studies.

Most transplant centres retain a small portion of stem cells harvested from a patient for quality purposes. Where possible, and with the participant’s consent, a small sample of these stem cells will be stored and shipped to the John van Geest Cancer Research Centre along with the other samples, for use in future ethically-approved research.

#### MRI scan

MRI scans will be undertaken at baseline, 24 and 48 weeks, according to standard clinical protocols using, at a minimum, a 1.5 T scanner and gadolinium contrast. Participants in the intervention group will also undergo an MRI scan at week 4, as part of the mechanistic analysis, to determine the early impact of the intervention on mucosal disease. Assessment for eligibility and recommencement of anti-TNF therapy at week 24 will be performed locally. However, sequences will also be recorded to a CD and the validated MaRIA score will be scored centrally by an investigator who is blind to the timing of assessment and treatment assignation, to confirm consistency.

### Procedures for assessing safety

The DMEC will assess safety of the HSCTlite mobilisation regimen after the first 10 patients, and subsequently at each DMEC meeting. Should the protocol fail to mobilise 2 × 10^6^ CD34^+^ cells/kg (haematopoietic stem and progenitor cells) in more than 10% patients, or if greater than 10% patients experience a disease flare up (increase in Harvey Bradshaw Index of > 30% from baseline associated with a rise in CRP) during mobilisation, a protocol amendment will be submitted to modify the mobilisation regimen for subsequent patients.

All AEs will be recorded in the CRF and will use the NCI classification of toxicity for 100-day safety post HSCT for assessment of grade.

All AEs, SAEs and Suspected Unexpected Serious Adverse Reactions (SUSARs) will be captured. All SAEs and SUSARs will be reported in accordance with the CTRU’s standard operating procedure. The Summary of Product Characteristics (SmPCs) for relevant products will be used as the reference safety information for reporting SAEs, and the PI is responsible for ensuring the assessment of expectedness and relatedness for all SAEs. Adverse events will be recorded from consent until the study closes. Although the timeframes for reporting SAEs differ between the RCT and the follow-up study, AEs will continue to be recorded throughout both elements of the study.

### Trial outcomes

#### Primary outcome

The primary outcome is treatment success at week 48, defined as mucosal healing (no endoscopic ulceration (SES CD ulcer sub score = 0, assessed by central readers blind to allocation and time of assessment)) without surgery or death.

Patients who do not complete the week 48 endoscopic assessment will be categorised as treatment failures.

The primary endpoint for the EBMT follow-up is the long-term safety of the HSCTlite regimen, for 4–7 years after the main ASTIClite RCT, as assessed by documentation of AEs.

### Secondary outcomes

#### Clinical endpoints

1. Clinical remission (CDAI < 150);
2. Steroid free clinical remission (CDAI < 150);
3. Clinical remission (Harvey Bradshaw Index ≤4);
4. Clinical remission (PRO2 – mean scores – abdominal pain ≤1, stool frequency ≤ 1.5);
5. Absolute CDAI at week 48;
6. Absolute SES CD at week 48;
7. Change in CDAI and SES CD between baseline and week 48;
8. Proportion of patients in complete endoscopic remission (SES CD score of 0);
9. Absolute MaRIA score at week 48.

#### Safety endpoints

1. Toxicity of chemotherapy using NCI CTCAE criteria version 4.03;
2. AEs and SAEs, including mortality.

#### Patient-reported endpoints

1. Disease specific quality of life using the IBDQ;
2. Disease specific quality of life using the IBD Control
3. Quality of life using the EQ-5D-5 L;
4. Health care resource utilisation questionnaire.

#### Exploratory secondary endpoints

1. Efficacy of re-introduction of anti-TNF therapy in patients with disease recurrence post-HSCT (change in CDAI at 6 weeks and change in SES CD at 22 weeks after initiation);
2. Safety of re-introduction of anti-TNF therapy in patients with disease recurrence post-HSCT;
3. Presence of any of the late side-effects of HSCT, documented through AEs.

### Secondary outcomes for EBMT follow-up study

The secondary endpoints for this study, for 4–7 years after stem cell transplant (and equivalent for the control arm) are:

1. Long term efficacy - for those receiving HSCT only;
2. Documentation of disease activity;
3. Requirement for further medical or surgical intervention;
4. Disease specific quality of life using the IBDQ questionnaire;
5. Disease specific quality of life using the IBD Control questionnaires;
6. Quality of life using the EQ-5D-5 L questionnaire;
7. Health care resource utilisation questionnaire.

## Sample size

The values in the calculations are based upon the endoscopic assessment post-HSCT reported in the ASTIC trial program [9, 10]. For the primary outcome, to detect a significant difference in the proportion of patients with absence of ulceration on endoscopic assessment of 35%, based on 50% in the HSCT group and no more than 15% in the control group, with 90% power at 5% significance level requires 62 patients in the HSCT group, and 31 in the control group. Therefore, 93 patients will be recruited at baseline, using 2:1 randomisation. Due to the nature of the condition, the design of the intervention and control group, the definition of the primary endpoint and our experience in the ASTIC trial, we anticipate a 6% drop out rate and will therefore recruit 99 patients (66 in the intervention group and 33 in the control group).

Based on experience in ASTIC, recruitment is anticipated to take 36 months. Patients will be recruited at 8 UK NHS Trusts, at an anticipated rate of 2.75 per month across all sites, or approximately 4 patients per site per year.

For the EBMT observational follow-up study, no additional sample size calculation has been performed; the study size is necessarily constrained by the number of patients recruited within the main RCT. However, allowing for drop-out, an expected sample size of *n* = 50 for the intervention arm at 4 years post-transplant will allow the prevalence of AEs to be estimated to a standard error of at most 7%.

## Data collection and management

The Sheffield CTRU’s bespoke database (Prospect) will be used to collect all trial data for the ASTIClite RCT. Site staff have access levels granted based on their study role, and evidence of appropriate training. Access is controlled by individual usernames and encrypted passwords. Site staff will enter data from source documents into Prospect, and electronic validation rules built into the system will ensure data queries are identified and resolved in a timely manner, on an ongoing basis.

Questionnaires are completed by participants onto paper copies and entered onto Prospect by site staff. Data will be stored and managed in accordance with CTRU Standard Operating Procedures (SOPs).

For the EBMT follow-up, the EBMT Registry database system MACRO (Elsevier) will be used for the capture and storage of participant data. This is a GDPR compliant web-based system, and access is controlled by usernames and encrypted passwords.

Participant confidentiality will always be respected; participants are identified only by study ID number alongside their data. Names and addresses/email addresses are only recorded for participants who consent to receive information about the trial.

AEs will be collected at each participant study visit. SAEs will be reported to the CTRU within 24 h of discovery, unless this is one of the exempt events defined in the protocol.

- Admissions to control symptoms of vomiting and diarrhoea, unless the condition requires admission to a high dependency or intensive care facility, is life-threatening or proves fatal (i.e. grade 4 or above, according to NCI CTCAE criteria);
- Admissions for supportive treatment during an episode of febrile neutropenia, unless this proves fatal or requires admission to a high dependency or intensive care facility (i.e. grade 4 or above, according to NCI CTCAE criteria);
- Admissions relating to myelosuppression unless the condition requires admission to a high dependency or intensive care facility, is life-threatening or proves fatal (i.e. grade 4 or above, according to NCI CTCAE criteria);
- Admissions relating to skin reactions and abnormal liver function tests caused by supportive care medications, unless the condition requires admission to a high dependency or intensive care facility, is life-threatening or proves fatal (i.e. grade 4 or above, according to NCI CTCAE criteria).

The Sheffield CTRU will be responsible for coordinating reporting to the Sponsor, REC and MHRA as required.

## Statistical analysis

### ASTIClite RCT

Analyses will be reported in line with the Consolidated Standards of Reporting Trials (CONSORT) 2010 Statement. Detailed descriptions of statistical analyses can be found in the ASTIClite statistical analysis plan. The trial statistician(s) will remain blinded throughout the study, but will be unblinded at database freeze, for analysis. The Senior Statistician within the CTRU will be unblinded to the treatment allocation throughout the trial, but will review and approve the statistical analysis plan before seeing any outcome data. Confidence intervals will be two-sided, 95% intervals and hypothesis tests will use a two-sided 5% level of significance. The primary analyses will be carried out using the intention to treat principle with data from all participants included in the analysis, including those who do not complete therapy and with participants analysed by the group to which they were randomised. Although the trial may terminate early on futility or safety grounds, no formal stopping rules are defined.

Analysis will be conducted in Stata version 14 or other validated statistical software as agreed by the study statisticians. Descriptive statistics for baseline values will be presented within each randomised group. These will include counts and percentages for binary and categorical variables and means and standard deviations, or medians with lower and upper quartiles, for continuous variables, along with minimum and maximum values and counts of missing values. There will be no tests of statistical significance or confidence intervals for differences between randomised groups on any baseline variable. Descriptive statistics will be used to summarise assessments of feasibility and acceptability in terms of recruitment, drop-out and completeness of therapy.

To test the primary hypothesis of a between group difference in the proportion of patients with absence of ulceration, we will estimate the proportions for each group. A mixed effects logistic regression will be used to estimate odds ratios for the disease remission in HSCTlite in comparison to conventional therapy. Baseline SES-CD ulcer subscore will be included as a fixed effect and study centre as a random effect. A number of sensitivity analyses will be carried out on the primary outcome, including assessing the impact of missing outcome data, adjustment for baseline predictors of missing outcome data and complier average causal effect (CACE) analysis.

For the secondary outcomes, analogous parametric regression models will be used as appropriate to the distributional form of the outcome, accounting for study centre, and the corresponding baseline assessment for the outcome under investigation where appropriate.

A secondary mediation analysis will investigate putative mediational factors using modern causal inference methods. This involves using parametric regression models to test for mediation of HSCT on treatment success through biomarkers. Analyses will adjust for baseline measures of the marker, and possible measured confounders.

### Mechanistic immunology

The complex datasets will be integrated, analysed and interpreted using established artificial neural network (ANN) and computational intelligence, machine-learning based approaches. Adaptions of existing neuro-fuzzy computational intelligence models [21] will be used to answer the questions posed. These approaches will provide important mechanistic insight into therapeutic responsiveness, underlying responsiveness to anti-TNF and events that are associated with patients becoming refractory to it after HSCT. Further details of the planned mechanistic analyses have been published elsewhere [22].

### Observational follow-up through the EBMT

The analysis will largely be descriptive, with incidence of AEs, markers of disease activity, quality of life and resource use summarised by time point and treatment arm. SAEs will be summarised both as the number and percentage in each year and by the incidence per person-year of follow-up, with the difference between groups summarised as incidence rate ratios and 95% confidence intervals. Continuous outcomes will be summarised using means, standard deviations and medians with interquartile ranges. Categorical variables will be summarised as counts and percentages and compared as the difference in percentages with 95% confidence intervals. Where appropriate, multivariable analyses will be performed using generalised linear models.

Patients who have HSCTlite after the end of the ASTIClite RCT will be included in the treatment arm for the long-term analysis for safety and efficacy. This means that the control group may reduce in size during the EBMT follow-up study, depending on how many patients receive transplant after the RCT.

## Discussion

CD is a distressing condition, and for many patients, there are no treatment options available to which the disease responds. Surgery is not always an option, and consequently some patients have a poor quality of life and are unable to work. The ASTIC trial suggested that HSCT could be beneficial to these patients, however, with the trial design and dosing regimen used in ASTIC, CD was not “cured”, and toxicity was unacceptably high for many patients.

ASTIClite aims to achieve these suggested beneficial outcomes, using a lower dose of the Investigational Medicinal Products (IMPs) in the HSCT regimen, using primary clinical outcomes that are designed to show beneficial effects, even if the patient still experiences CD recurrence.

Furthermore, ASTIClite will assess the clinical efficacy and long-term impact of lower doses of cyclophosphamide in the mobilisation of PBSCs followed by a reduced intensity fludarabine, cyclophosphamide and ATG-based conditioning regimen in patients with active CD refractory to biologic therapies. Embedded mechanistic studies will assess the timeline of response, immune reconstitution after HSCT and the mechanism by which HSCT alleviates disease and/or restores anti-TNF responsiveness in this previously refractory group.

### Current status

The current protocol for the RCT is version 6.1, 05.03.2019, and for the EBMT follow up study version 3.1, 01.10.2018. The ASTIClite RCT and EBMT follow up study both started recruiting in June 2018. Recruitment is expected to finish in March 2021, with completion of the RCT in March 2022, and the EBMT follow up study in March 2026. We anticipate that results from the RCT will be available in late 2022.

### Monitoring

Conduct of the study is overseen by three committees; an independent Trial Steering Committee (TSC) to oversee overall trial conduct, an independent Data Monitoring and Ethics Committee (DMEC) to monitor safety of trial participants, and a Trial Management Group (TMG), responsible for the day-to-day running of the trial. The TMG is made up of the site investigators, collaborators, study management team and statisticians, and clinical members will also discuss eligibility of all potential trial participants, to confirm suitability for randomisation. Each committee has a charter or terms of reference, which outlines the roles and responsibilities in full.

Remote monitoring will regularly review trial data, missing data and data queries for timely resolution. In addition, CTRU will undertake monitoring visits at each investigator site before, during and after the trial. Central, remote monitoring will be used alone for the observational follow-up study, after the end of the RCT.

### Ethics and dissemination

The ASTIClite RCT was given a favourable ethical opinion from the London – Chelsea NHS Research Ethics Committee (reference: 17/LO/1690), and the EBMT follow-up study from North West – Greater Manchester East NHS Research Ethics Committee (reference: 17/NW/1669). All protocol amendments will be approved by the relevant ethics committee, and regulatory authority as applicable, before being notified to trial investigators.

Trial results will be disseminated in peer-reviewed scientific journals and clinical and academic conferences. A lay summary of the results will be made available on the study website at the end of the trial and will be disseminated to participants who consented to receive information about the trial. The main trial results will also be published on the NIHR EME journal website.

### Patient and public involvement

Patient-facing documents were reviewed by two patients who took part in the ASTIC trial, to ensure ease of understanding, readability, format and to address any concerns with the study design from a patient point of view. Any significant amendments to patient facing documents will be discussed with this patient panel prior to implementation.

In addition, two patient representatives are members of the TSC, for ongoing patient involvement in the management of the study, and a further patient perspective on major decisions affecting trial processes or conduct.

## Appendix

**Table 3:** Study assessment schedule

	Screening^1^	Baseline		Week 4 (HSCT only)	Week 8	Week 14	Week 24	Week 32	Week 40	Week 48
Assessments										
Eligibility assessment	✓	✓	HSCT procedure (intervention) or continuation on current treatment (control)							
Consent	✓									
Standard Pre-HSCT work (including chest x-ray and MUGA scan)	✓									
Serology for HBV, HCV, HIV	✓									
Demographics	✓									
Medication history	✓									
Concomitant medications	✓	✓		✓	✓	✓	✓	✓	✓	✓
Adverse events				✓	✓	✓	✓	✓	✓	✓
General Medical History	✓									
History of CD	✓									
General Physical Examination	✓	✓			✓	✓	✓	✓	✓	✓
Urinalysis	✓	✓								
Pregnancy test	✓	✓								
Smoking History	✓									
Crohn’s Disease Activity Index (CDAI)	✓	✓			✓	✓	✓	✓	✓	✓
Harvey Bradshaw Index	✓	✓			✓	✓	✓	✓	✓	✓
Karnofsky Performance Status	✓									✓
Patient Reported Outcome 2 questionnaire (PRO2)	✓				✓	✓	✓	✓	✓	✓
Ileo-colonoscopy (Simple Endoscopic Score for Crohn’s Disease (SES CD)) / endoscopic assessment	✓						✓			✓
Biopsies^2^	✓						✓			✓
MRI Intestine	✓			✓			✓			✓
MRI Pelvis	✓									
Routine Clinical Care blood test		✓			✓	✓	✓	✓	✓	✓
Serum^3^		✓			✓	✓	✓	✓	✓	✓
Whole Blood^3^		✓			✓	✓	✓	✓		✓
Peripheral Blood mononuclear cells (PBMCs)^3^		✓			✓	✓	✓	✓		✓
Stool sample^3^		✓			✓	✓	✓	✓		✓
Inflammatory Bowel Disease Questionnaire (IBDQ)	✓				✓	✓	✓	✓	✓	✓
Inflammatory Bowel Disease Control Questionnaire (IBD-Control)	✓				✓	✓	✓	✓	✓	✓
100 day safety (collection of Adverse Events for transplant endpoint)						✓				
EQ-5D-5 L	✓				✓	✓	✓	✓	✓	✓
Work Productivity and Activity Impairment questionnaire (WPAI)	✓				✓	✓	✓	✓	✓	✓
Health Care Resource Use Questionnaire	✓				✓	✓	✓	✓	✓	✓
Patient Global Impression of Change (PGIC)										✓
For participants in HSCTlite arm only:										
JACIE and HTA recommended routine tests		✓	HSCT procedure (intervention) or continuation on current treatment (control)							
Anti-TNF therapy initiated (if required)							✓			
Adherence to re-vaccination policy						✓	✓	✓	✓	✓

## Abbreviations

AE(s): Adverse events
ATG: Anti-thymocyte globulin
CD: Crohn’s disease
CDAI: Crohn’s Disease Activity Index
CTRU: Clinical Trials Research Unit
DMEC: Data Monitoring and Ethics Committee
EBMT: European Society for Blood and Marrow Transplantation
EQ-5D: EuroQol Five Dimensions Questionnaire
G-CSF: Granulocyte-colony stimulating factor
HBI: Harvey Bradshaw Index
HSCT: Haematopoetic stem cell transplantation
IBD: Inflammatory bowel disease
IBDQ: Inflammatory Bowel Disease Questionnaire
IMP(s): Investigational Medicinal Product(s)
JACIE: Joint Accreditation Committee – ISCT & EBMT
MaRIA: Magnetic Resonance Index of Activity
MDT: Multidisciplinary team
MHRA: Medicines and Healthcare products Regulatory Agency
NCI CTCAE: National Cancer Institute Common Terminology Criteria for Adverse Events
NICE: National Institute for Health and Care Excellence
NIHR: National Institute for Health Research
NIMP: Non-investigational medicinal product
PBSC(s): Peripheral blood stem cells(s)
PI: Principal investigator
REC: Research Ethics Committee
SAE(s): Serious adverse event(s)
SES-CD: Simple Endoscopic Score for Crohn’s Disease
SUSAR(s): Suspected Unexpected Serious Adverse Reaction(s)
TMG: Trial Management Group
TSC: Trial Steering Committee
WPAI: Work Productivity and Activity Impairment
